# Stability of Intraocular Lens With Different Haptic Design: A Swept-Source Optical Coherence Tomography Study

**DOI:** 10.3389/fmed.2021.705873

**Published:** 2021-09-08

**Authors:** Zixuan Xiao, Geng Wang, Miaoru Zhen, Zifeng Zhao

**Affiliations:** Joint Shantou International Eye Center of Shantou University and The Chinese University of Hong Kong, Shantou, China

**Keywords:** intraocular lens, tilt, decentration, haptic, optical coherence tomography

## Abstract

**Purpose:** To investigate the stability of intraocular lens (IOLs) with different haptics by swept-source anterior-segment optical coherence tomography (AS-OCT).

**Methods:** Sixty-eight eyes from 55 patients received the implantation of Rayner 920H (Closed C-loop Group), Zeiss 509M (Plate Group) or Lenstec SOFTEC HD (C-loop Group) IOLs. The tilt and decentration of IOLs were evaluated using AS-OCT at least 1 month postoperatively.

**Results:** Mean decentration and tilt of IOLs were 0.18 ± 0.12 mm (range 0.02 to 0.59 mm) and 5.63 ± 1.65° (range 2.2 to 9.6°) respectively. Decentration was significantly smaller in the plate haptic group (0.12 ± 0.06 mm) as compared to the C-loop group (0.22 ± 0.13 mm, *P* = 0.02). The tilt of IOL was also significantly smaller in the plate haptic group (4.96 ± 0.89°) as compared to the C-loop group (6.28 ± 1.83°, *P* = 0.01). There was marginal difference between the Closed C-loop group (5.52 ± 1.74°) and C-loop group (6.28 ± 1.83°, *P* = 0.07).

**Conclusions:** The Plate-haptic IOLs should have better stability for the decentration and tilt than the C-loop design IOLs.

## Introduction

Cataract is the leading cause of reversible blindness in the world (1, 2). Phacoemulsification with foldable intraocular lens (IOLs) implantation is still the main treatment for cataract (3). There are many different types of commercially available IOLs (4–6). The positions of IOLs are crucial for visual outcome after cataract surgeries. Any decentration and tilt of IOLs would induce wavefront aberrations and affect visual performance (7).

The maintenance of IOL stability relies on the support of the haptics in the capsular bag (8). Previous study based on Scheimpflug imaging demonstrated that the one-piece IOLs show better stability than the three-piece IOLs (9–11). Using OPD-Scan III aberrometer (Nidek Co, Ltd., Gamagori, Japan), another study reported that the Plate-haptic IOLs show better stability than the C-loop IOLs in myopic eyes (8). Both studies measured decentration and tilt of IOLs manually.

IOLs with Plate-haptics, C-loop or Closed C-loop are widely used in clinical practice. It is not clear whether these IOLs with different haptics would have different performance in stability. The CASIA2 (CASIA2, TOMEY, Nagoya, Japan) is a new generation anterior segment optical coherence tomography (AS-OCT) with a 1,310 nm swept-source laser (12). The lens analysis mode of CASIA2 could automatically measure and analyze the IOL decentration and tilt using the corneal topographic axis with high repeatability and reproducibility (12, 13). To our knowledge, no study has used CAISA2 to analyzed tilt and decentration of these kinds of IOLs. Herein this study aimed to investigate the decentration and tilt of IOLs with different haptics by the swept-source AS-OCT.

## Materials and Methods

### Study Design and Ethical Approval

This study was a retrospective study. It has been approved by the Human Medical Ethics Committee of the Joint Shantou International Eye Center (JSIEC) of Shantou University and the Chinese University of Hong Kong, which was in accordance with the tenets of the Declaration of Helsinki.

### Subjects

Sixty-eight eyes from 55 Chinese adults who had underwent cataract surgeries were involved. All patients were followed up at JSIEC from May 2019 to January 2020. All subjects received complete ophthalmic examinations, including visual acuity, intraocular pressure (IOP), dilated fundus stereoscopic examination. The exclusion criteria were as follows: (1) age <18; (2) follow-up time <1 month; (3) history of eye surgeries other than cataract surgery; (4) history of complications during or after cataract surgery; (5) severe anterior capsule contraction; (6) angle closure glaucoma; (7) high myopia or axial length (AL) ≥ 26 mm.

### Intraocular Lenses

All 68 eyes from 55 Chinese adult study subjects received routine phacoemulsification and IOL implantation in the capsular bag. Closed C-loop IOLs (Rayner 920H) were implanted in 25 eyes of 22 subjects. C-loop IOLs (Lenstec Softec HD) were implanted in 24 eyes of 19 subjects. Plate-haptics IOLs (Zeiss 509M) were implanted in 19 eyes of 14 subjects. The information of IOLs used in the current study was shown in Table 1. Rayner 920H has an overall diameter of 12.5 and 6.25 mm of optical diameter with Closed C-loop haptic design. Lenstec Softec HD has an overall diameter of 12 mm and 5.75 mm of optical diameter with C-loop haptic design. Zeiss 509M has an overall diameter of 11 and 6 mm of optical diameter with Plate-haptic design. All these IOLs were made of hydrophilic acrylic material and 0° in angulation.

**Table 1:** Characteristics of the three types of intraocular lens.

	**Closed-C loop**	**C-loop**	**Plate-haptic**
Material	Hydrophilic acrylic	Hydrophilic acrylic	Hydrophilic acrylic
Total diameter (mm)	12.5	12	11
Optic diameter (mm)	6.25	5.75	6
Haptics style	Closed C-loop	C-loop	Plate
Angulation	0°	0°	0°

### Anterior Segment Optical Coherence Tomography Imaging

**Figure 1:**
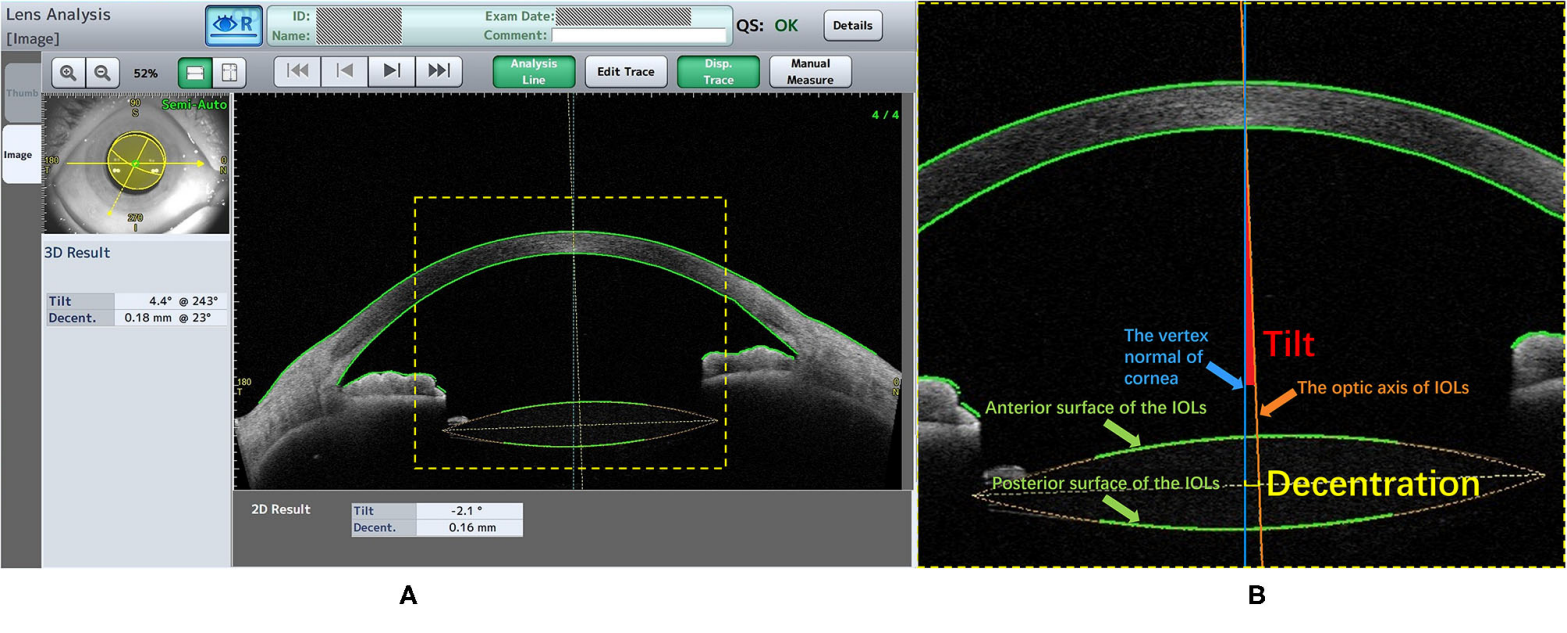
The lens analysis of CASIA2 examination. **(A)** The information of patient and the Quality Score (QS) were shown above, left window showed the 3D result and right window showed one of eight anterior segment images. The 3D result contained the value and the azimuth of the IOLs tilt and decentration. **(B)** The Green line (Pointed by the Green arrow): the trace lines on the anterior and posterior surface of the IOLs; The Orange line (Pointed by the Orange arrow): the IOLs optic axis which was defined as a perpendicular line with the center of equator circle of IOLs; The Blue line (Pointed by the Blue arrow): the vertex normal of cornea. The decentration (The short yellow line) was defined as a distance between vertex normal and the center of equator circle of lens. The tilt (The red area) was defined as an angle between vertex normal and optic axis.

**Figure 2:**
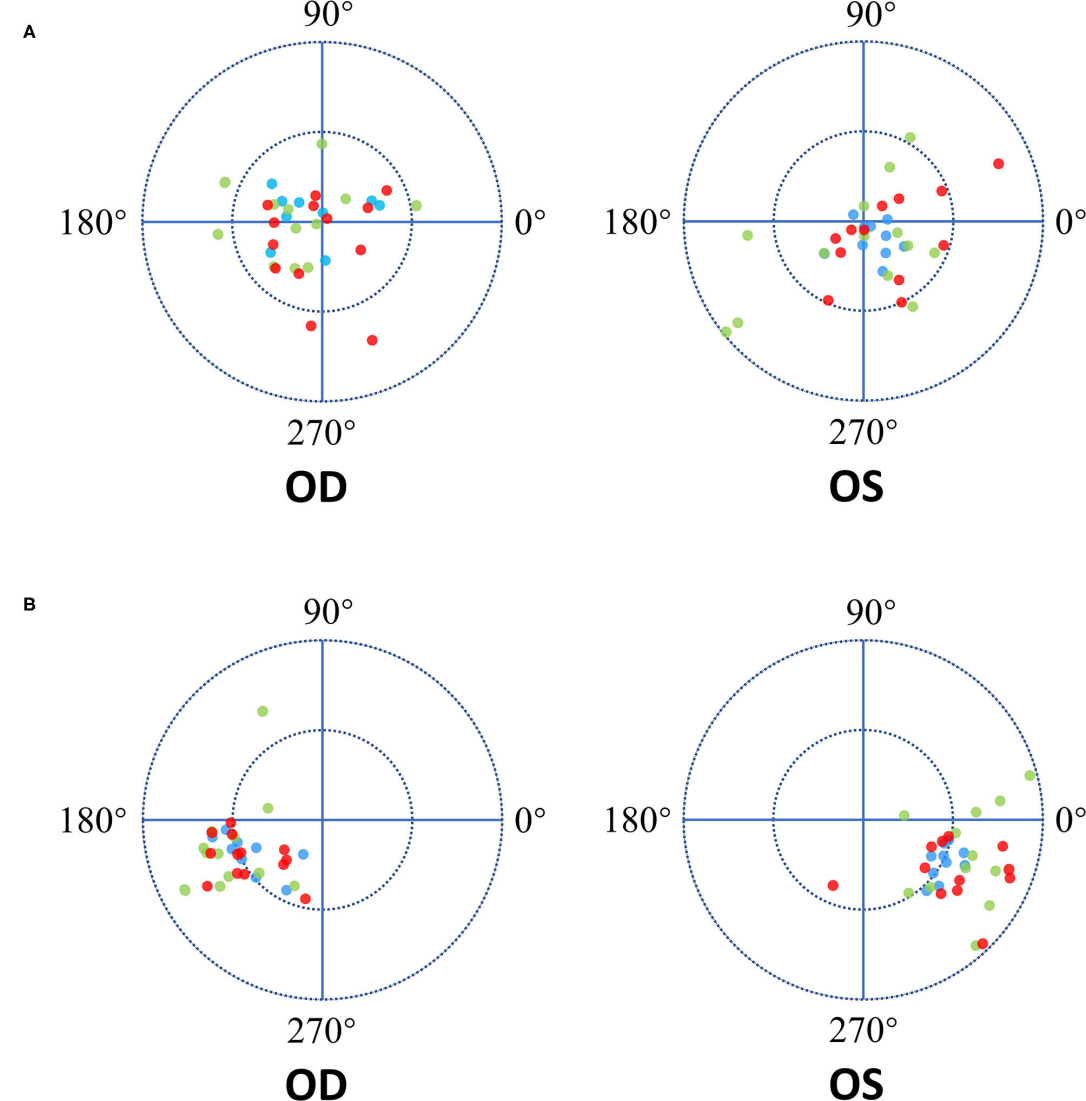
The Diagram of IOL decentration and tilt direction. The length between the dot and the origin of coordinates is the decentration and tilt magnitude. The azimuth represented the orietation of IOL tilt and decentration in degree. Zero-degree was in the nasal side of the patient's right eye and in the temporal side of the patient's left eye. OD represent the right eye of patient while OS represent the left eye. The red dots represent the Closed C-loop IOLs, the green dots represent the C-loop IOLs and the blue dots represent the Plate IOLs. **(A)** Each ring = 0.3mm. The blue dots showed more concentrated on the origin of coordinates in both eyes. And there was not obvious tendency of the IOLs decentration direction in both eyes. **(B)** Each ring = 5°. The tendency of IOLs tilt direction was toward the inferotemporal direction in the both eyes.

All studied eyes were dilated using a mixture of 0.5% tropicamide and 0.5% phenylephrine hydrochloride (Mydrin P, Santen, Osaka, Japan) at 30 min before CASIA2 examination. All examinations were performed by a single experienced operator. “Post-op Cataract” was selected in Exam Protocol Selection. Trace Line mode was used with “Semi-Auto”. Eight anterior segment images were obtained from 8 scans (0 to 180°, 90 to 270°, 23 to 203°, 113 to 293°, 45 to 225°, 135 to 315°, 68 to 248°, and 158 to 338°). Three-dimensional anterior segment was reconstructed. The positions of IOLs were assessed automatically by the lens analysis mode in the device-specific software. IOL decentration was defined as a distance between vertex normal of the cornea and the center of equator circle of IOLs, while IOL tilt was defined as a tilt between vertex normal of the cornea and the optic axis of IOLs (Figure 1). Moreover, the device-specific software can export the azimuth which represented the orietation of IOL tilt and decentration in degree. The azimuth can show the direction of maximal IOL tilt and decentration, which was indicated graphically by a coordinate system (Figure 2). If the anterior and posterior surface of the IOLs were not correctly traced or the Quality Score (QS) was not satisfactory, the scan would be repeated. The data of lOLs tilt and decentration was collected from the report generated by CAISA2.

### Statistical Analysis

Statistical analysis was performed with commercially available software (SPSS ver. 26.0; SPSS Inc, Chicago, IL). Data distribution for normality was checked using Shapiro–Wilk's test. According to the data normality of the groups, one-way analysis of variance (ANOVA) or Kruskal-Wallis H test was used to compare the age, AL, IOL decentration and tilt among different groups. Multiple testing correction between groups was adjusted by the *post-hoc* test. χ^2^ test was used for the comparisons on gender and laterality. *P* < 0.05 was considered as statistical significant.

## Results

Sixty-eight eyes from 55 study subjects were included in the analysis. There were 25 eyes in the Closed C-loop Group, 24 eyes in the C-loop Group and the 19 eyes in the Plate Group. The demographics of the study subjects were shown in Table 2. The mean age of the Closed C-loop, C-loop and Plate groups was 63.84 ± 15.02 years, 68.08 ± 9.36 years and 63.47 ± 6.18 years respectively. Mean AL in the Closed C-loop, C-loop and Plate groups was 23.68 ± 0.88 mm, 23.73 ± 0.94 mm and 23.51 ± 0.70 mm respectively. There was no statistically significant differences in age, AL, gender and laterality among the three studied groups.

**Table 2:** Demographics of study subjects implanted with different haptic design of intraocular lenses.

	**Closed C-loop** **(*n* = 25)**	**C-loop** **(*n* = 24)**	**Plate** **(*n* = 19)**	***P***
^†^Age (years, mean ± SD)	63.84 ± 15.02	68.08 ± 9.36	63.47 ± 6.18	0.17
^†^AL (mm, mean ± SD)	23.68 ± 0.88	23.73 ± 0.94	23.51 ± 0.70	0.75
^*^Eye (right/left)	13/12	12/12	9/10	0.98
^*^Gender (male/female)	8/17	11/13	10/9	0.37

*AL, axial length*.

* *χ^2^ test*.

† *Kruskal-Wallis H test*.

### Intraocular Lens Decentration

Mean decentration for all types of IOLs was 0.18 ± 0.12 mm (range 0.02 to 0.59 mm). There were significant differences among the three studied groups in decentration (ANOVA, *P* = 0.02). The IOL decentrations of the three studied groups were shown in Table 3 and Figure 2. Mean decentration of the Closed C-loop, C-loop and Plate groups was 0.19 ± 0.12 mm (range 0.02 to 0.49 mm), 0.22 ± 0.13 mm (range 0.02 to 0.59 mm) and 0.12 ± 0.06 mm (range 0.02 to 0.21 mm) respectively. Decentration was significantly smaller in the plate haptic group (0.12 ± 0.06 mm) as compared to the C-loop group (0.22 ± 0.13 mm, Bonferroni-Dunn test, *P* = 0.02). There was no statistically significant difference between the Plate and Closed C-loop groups in decentration, and no significant difference in decentration was found between the Closed C-loop and C-loop groups. Decentration for IOLs was toward each quadrant without obvious tendency (mean: 155.50° for right eyes and 87.50° for left eyes). For the right eyes, mean IOL decentration azimuth of the Closed C-loop, C-loop and Plate groups was 159.08 ± 103.33° (range 17 to 324°), 155.92 ± 73.56° (range 10 to 253°) and 135.33 ± 83.94° (range 16 to 275°) respectively. For the left eyes, mean IOL decentration azimuth of the Closed C-loop, C-loop and Plate groups was 186.33 ± 122.14° (range 21 to 343°), 226.08 ± 105.65° (range 61 to 341°) and 249.40 ± 102.89° (range 4 to 329°) respectively. This tendency was not affected by the haptic design (right eyes: Kruskal-Wallis H test, *P* = 0.51; left eyes: Kruskal-Wallis H test, *P* = 0.40).

**Table 3:** Intraocular lens stability with different haptics.

	**Closed C-loop** **(** ***n*** **= 25)**	**C-loop** **(** ***n*** **= 24)**	***P***	**Closed C-loop** **(** ***n*** **= 25)**	**Plate** **(** ***n*** **= 19)**	***P***	**C-loop** **(** ***n*** **= 24)**	**Plate** **(** ***n*** **= 19)**	***P***
Decentration (mm, mean ± SD)	0.19 ± 0.12	0.22 ± 0.13	1.00^*^	0.19 ± 0.12	0.12 ± 0.06	0.10^*^	0.22 ± 0.13	0.12 ± 0.06	0.02^*^
Tilt (degree, mean ± SD)	5.52 ± 1.74°	6.28 ± 1.83°	0.07^†^	5.52 ± 1.74°	4.96 ± 0.89°	0.29^†^	6.28 ± 1.83°	4.96 ± 0.89°	0.01^†^

* *ANOVA*.

† *Kruskal-Wallis H test*.

### Intraocular Lens Tilt

Mean tilt for all IOLs was 5.63 ± 1.65° (range 2.20 to 9.60°). There were significant differences among the three studied groups in IOL tilt (Kruskal-Wallis H test, *P* = 0.02). IOL tilts of the three studied groups were shown in Table 3 and Figure 2. Mean IOL tilt of the Closed C-loop, C-loop and Plate groups was 5.52 ± 1.74° (range 2.70 to 9.40°), 6.28 ± 1.83° (range 2.30 to 9.60°) and 4.96 ± 0.89° (range 2.20 to 6.20°) respectively. The Plate Group showed significantly smaller IOL tilt (4.96 ± 0.89°) than the C-loop Group (6.28 ± 1.83°, Bonferroni-Dunn test, *P* = 0.01). Ther was marginal difference in IOL tilt between the Closed C-loop Group (5.52 ± 1.74°) and C-loop Group (6.28 ± 1.83°, Bonferroni-Dunn test, *P* = 0.07), and no significant difference was found between the Plate Group and Closed C-loop Group in IOL tilt. Mean IOL tilt direction was toward the inferotemporal direction (204.74° for right eyes and 290.85° for left eyes), and both eyes presented a mirror symmetry relationship. For the right eyes, mean IOL tilt azimuth of the Closed C-loop, C-loop and Plate groups was 209.92 ± 20.68° (range 182 to 258°), 195.83 ± 31.13° (range 119 to 247°) and 209.11 ± 21.26° (range 186 to 243°) respectively. For the left eyes, mean IOL tilt azimuth of the Closed C-loop, C-loop and Plate groups was 325.50 ± 28.33° (range 244 to 352°), 221.58 ± 158.12° (range 15 to 305°) and 332.40 ± 11.30° (range 312 to 347°) respectively. This tendency was not affected by the haptic design (right eyes: Kruskal-Wallis H test, *P* = 0.53; left eyes: Kruskal-Wallis H test, *P* = 0.14).

## Discussion

In the current study, the stability of three different types of IOLs with C-loop, Closed C-loop and Plate-haptics was evaluated with CASIA 2 AS-OCT. The Plate-haptic IOLs were found to have the smallest decentration and tilt among the three studied groups. The C-loop IOLs were found to have the largest decentration and tilt among the three studied groups.

The positions of IOLs are crucial for postoperative visual performance. Any decentration or tilt of IOLs could induce wavefront aberrations and lower visual performance (7). Previous studies indicated that decentration would induce coma, astigmatism and defocus (14). It has been (15) reported that visual function of aspheric IOLs is worse than spherical IOLs when the tilt is more than 7° or when decentration is more than 0.4 mm. Another study (14) based on the Liou-Brennan model eye found that the aspherical IOLs are more sensitive to decentration and tilt than spherical IOLs. Similar results were also found in additional study (16). It has been suggested that (7), 0.3 mm decentered IOL could cause hyperopic shift of less than 0.11 D, while IOL tilt of 5° could result in a myopic shift of up to 0.25 D. IOL tilt could be more clinically relevant than decentration.

In previous studies based on Purkinje method and Scheimpflug method (8, 14, 17), mean decentration was 0.30 ± 0.16 mm (range 0.00 to 1.09 mm), and mean tilt was 2.62 ± 1.14° (range 0.20 to 8.17°). The range of tilt measured by Purkinje method was limited (18). The Scheimpflug method suffered from the optical distortion which could influence on the measurement of tilt and decentration (17, 19). Both Purkinje method and Scheimpflug method needed manual marking and relied on pupil as reference axis. It has been (13) shown that the methods relied on pupil as reference axis could be affected by the shape and location of the pupil. Instead, the corneal topographic axis should be the better choice of the reference axis. The current study used CASIA2 to measure the IOL positions. CASIA2 is a new-generation AS-OCT with a 1,310-nm swept-source laser wavelength (12). It could automatically measure the IOL position using the corneal topographic axis with high repeatability and reproducibility (13, 20). Mean decentration and tilt of IOLs in the current study were 0.18 ± 0.12 mm (range 0.02 to 0.59 mm) and 5.63 ± 1.65° (range 2.20 to 9.60°), which were within the range of previous studies. Compared to the previous studies, this study reported relatively smaller mean decentration and larger mean tilt. The discrepancy of the current study with previous studies could be due to different methods with different algorithms and reference axes. The current study found an obvious tendency of IOLs tilt toward the inferotemporal direction relative to the corneal topographic axis, and there was a mirror symmetry relationship in both eyes. This result was consistent with the previous study using IOLMater700(21). It was (13) found that the crystalline lens is tilting toward the inferotemporal direction. This tilting tendency could be due to the normal physiological structure of the eyeball. In addition, our study found that the tendency of IOL tilt showed no relationship with the haptics design of IOLs.

**Figure 3:**
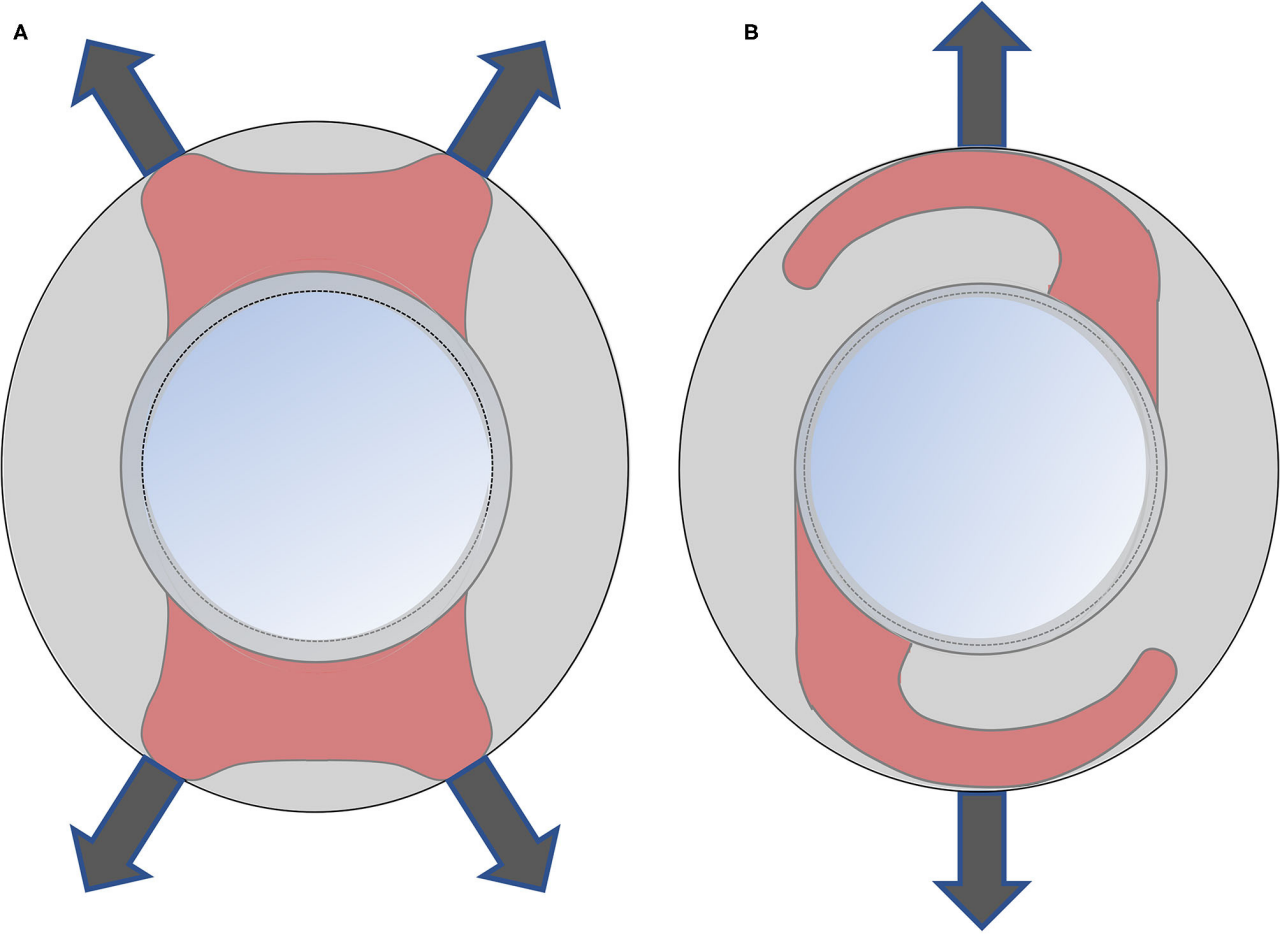
Comparison of Plate-haptics and C-loop IOL. The gray area is the capsule. The red area is the area of haptics covered by the capsule. **(A)** The Plate-haptics IOLs can get great support from the capsular bag through the four corners and more area of haptics can be covered by capsule compared to C-loop IOLs. **(B)** The C-loop IOLs has only two support points against capsule and there is a large gap between the optic and haptics.

The current study found that the Plate-haptics IOLs have the smallest absolute mean of the decentration and tilt among the three studied IOLs. The Plate-haptics IOLs showed better stability than the C-loop IOLs. There was no significant difference between the Plate and Closed C-loop groups for IOL decentration and tilt. No significant difference was also found between Closed C-loop Group and C-loop Group for IOL decentration and tilt. Previous study reported that the Plate-haptics IOLs have better stability than the C-loop IOLs in myopic eyes (8), which is consistent with the current study. The Plate-haptics IOLs could achieve greater support from the capsular bag through the four corners of the IOLs. Moreover, there is no gap between the optic and haptics in Plate-haptics IOLs, which is different from the C-loop IOLs. There is larger area of haptics that could be covered by the capsule. Moreover, the C-loop IOLs have only two support points against the capsule. More decentration could be developed when the capsule bag has asymmetric fibrosis (Figure 3). As a result, the Plate-haptics IOLs have less decentration and tilt than the C-loop IOLs. The current study also found that there was marginal difference between the Closed C-loop and C-loop group for IOL tilt. We postulated that the Closed C-loop design could have better stability than the C-loop in IOL tilt. The Closed C-loop has outer and inner haptics, while the C-loop has two single haptics. When there is capsule contraction, the outer haptics would first hold against the contraction. When the contraction becomes more severe, the outer haptics and inner haptics would be in contact, and the resistance would become greater to maintain the position of IOLs.

There were several limitations in the current study. First, the sample size was relatively small. Second, hydrophobic C-loop IOLs was not involved in the current study. Hydrophobic C-loop IOLs has been widely used in clinical practices and could have different performance in IOL stability.

In summary, this study evaluated the decentration and tilt of three different IOLs with C-loop, Closed C-loop and Plate-haptics. The Plate-haptics IOLs showed better stability for the decentration and tilt than the C-loop design IOLs. No significant difference in stability was found between Plate-haptics and Closed C-loop IOLs. Further studies with larger sample size are warranted to confirm the preferred design of haptics for IOLs.

## Data Availability Statement

The raw data supporting the conclusions of this article will be made available by the authors, without undue reservation.

## Ethics Statement

The study was approved by the Ethics Committee of the Joint Shantou International Eye Center (JSIEC), Shantou, China. Written informed consent for participation was not required for this study in accordance with the national legislation and the institutional requirements.

## Author Contributions

GW contributed to administrative, research conception, technical, and material supports. ZX contributed to research conception, data collection, analysis, and writing of the manuscript. MZ and ZZ contributed to data collection. All authors saw and approved the final version.

## Funding

This study was supported by Guangdong Medical Research Foundation (Grant No. A2016514), Shantou Municipal Science and Technology Project (Grant No. 190917155269927), 2020 Li Ka Shing Foundation Cross-Disciplinary Research Grant (Project Number: 2020LKSFG06B), and Grant for Key Disciplinary Project of Clinical Medicine under the Guangdong High-level University Development Program, China.

## Conflict of Interest

The authors declare that the research was conducted in the absence of any commercial or financial relationships that could be construed as a potential conflict of interest.

## Publisher's Note

All claims expressed in this article are solely those of the authors and do not necessarily represent those of their affiliated organizations, or those of the publisher, the editors and the reviewers. Any product that may be evaluated in this article, or claim that may be made by its manufacturer, is not guaranteed or endorsed by the publisher.
